# Chemical Composition and Antioxidant, Antimicrobial, and Anti-Inflammatory Properties of *Origanum compactum* Benth Essential Oils from Two Regions: In Vitro and In Vivo Evidence and In Silico Molecular Investigations

**DOI:** 10.3390/molecules27217329

**Published:** 2022-10-28

**Authors:** Samiah Hamad Al-Mijalli, Nidal Naceiri Mrabti, Hayat Ouassou, Ryan A. Sheikh, Hamza Assaggaf, Saad Bakrim, Emad M. Abdallah, Mohammed Merae Alshahrani, Ahmed Abdullah Al Awadh, Learn-Han Lee, Yusra AlDhaheri, Amirhossein Sahebkar, Gokhan Zengin, Ammar A. Attar, Abdelhakim Bouyahya, Hanae Naceiri Mrabti

**Affiliations:** 1 Department of Biology, College of Sciences, Princess Nourah Bint Abdulrahman University, P.O. Box 84428, Riyadh 11671, Saudi Arabia; 2 Computer Chemistry and Modeling Team, Laboratory of Materials, Modeling and Environmental Engineering (LIMME), Faculty of Sciences Dhar El Mehraz, Sidi Mohamed Ben Abdellah University (USMBA), BP 1796, Atlas, Fez 30000, Morocco; 3 Faculty of Sciences, University Mohammed First, Boulevard Mohamed VI, BP 717, Oujda 60000, Morocco; 4 Biochemistry Department, Faculty of Science, King Abdulaziz University, Jeddah 21589, Saudi Arabia; 5 Department of Laboratory Medicine, Faculty of Applied Medical Sciences, Umm Al-Qura University, Makkah 21955, Saudi Arabia; 6 Geo-Bio-Environment Engineering and Innovation Laboratory, Molecular Engineering, Biotechnologies and Innovation Team, Polydisciplinary Faculty of Taroudant, Ibn Zohr University, Agadir 80000, Morocco; 7 Department of Science Laboratories, College of Science and Arts, Qassim University, Ar Rass 51921, Saudi Arabia; 8 Department of Clinical Laboratory Sciences, Faculty of Applied Medical Sciences, Najran University, P.O. Box 1988, Najran 61441, Saudi Arabia; 9 Novel Bacteria and Drug Discovery Research Group (NBDD), Microbiome and Bioresource Research Strength (MBRS), Jeffrey Cheah School of Medicine and Health Sciences, Monash University Malaysia, Bandar Sunway 47500, Malaysia; 10 Department of Biology, College of Science, United Arab Emirates University, Al Ain P.O. Box 15551, United Arab Emirates; 11 Biotechnology Research Center, Pharmaceutical Technology Institute, Mashhad University of Medical Sciences, Mashhad 9177948954, Iran; 12 Department of Biotechnology, School of Pharmacy, Mashhad University of Medical Sciences, Mashhad 9177948954, Iran; 13 Biochemistry and Physiology Research Laboratory, Department of Biology, Faculty of Science, Selcuk University, 42130 Konya, Turkey; 14 Laboratory of Human Pathologies Biology, Department of Biology, Faculty of Sciences, Mohammed V University in Rabat, Rabat 10106, Morocco; 15 Laboratory of Pharmacology and Toxicology, Bio Pharmaceutical and Toxicological Analysis Research Team, Faculty of Medicine and Pharmacy, Mohammed V University in Rabat, BP 6203, Rabat 10000, Morocco; 16 Laboratoires TBC, Faculty of Pharmaceutical and Biological Sciences, B.P. 8359006 Lille, France

**Keywords:** *Origanum compactum*, essential oils, anti-inflammatory, tyrosinase, antibacterial action

## Abstract

The purposes of this investigatory study were to determine the chemical composition of the essential oils (EOs) of *Origanum compactum* from two Moroccan regions (Boulemane and Taounate), as well as the evaluation of their biological effects. Determining EOs’ chemical composition was performed by a gas chromatography–mass spectrophotometer (GC-MS). The antioxidant activity of EOs was evaluated using free radical scavenging ability (DPPH method), fluorescence recovery after photobleaching (FRAP), and lipid peroxidation inhibition assays. The anti-inflammatory effect was assessed in vitro using the 5-lipoxygenase (5-LOX) inhibition test and in vivo using the carrageenan-induced paw edema model. Finally, the antibacterial effect was evaluated against several strains using the disk-diffusion assay and the micro-dilution method. The chemical constituent of *O. compactum* EO (OCEO) from the Boulemane zone is dominated by carvacrol (45.80%), thymol (18.86%), and α-pinene (13.43%). However, OCEO from the Taounate zone is rich in 3-carene (19.56%), thymol (12.98%), and o-cymene (11.16%). OCEO from Taounate showed higher antioxidant activity than EO from Boulemane. Nevertheless, EO from Boulemane considerably inhibited 5-LOX (IC_50_ = 0.68 ± 0.02 µg/mL) compared to EO from Taounate (IC_50_ = 1.33 ± 0.01 µg/mL). A similar result was obtained for tyrosinase inhibition with Boulemane EO and Taounate EO, which gave IC_50s_ of 27.51 ± 0.03 μg/mL and 41.83 ± 0.01 μg/mL, respectively. The in vivo anti-inflammatory test showed promising effects; both EOs inhibit and reduce inflammation in mice. For antibacterial activity, both EOs were found to be significantly active against all strains tested in the disk-diffusion test, but *O. compactum* EO from the Boulemane region showed the highest activity. Minimum inhibitory concentrations (MICs) and minimum bactericidal concentrations (MBCs) for *O. compactum* EO from the Boulemane region ranged from 0.06 to 0.25% (*v*/*v*) and from 0.15 to 0.21% (*v*/*v*) for *O. compactum* from the Taounate region. The MBC/MIC index revealed that both EOs exhibited remarkable bactericidal effects.

## 1. Introduction

Historically, plant-based medications have been the only therapies used by all traditional medical systems for thousands of years [1]. The pharmaceutical industry has been very successful throughout the 20th century with synthetic chemistry, which has proven to be a very effective treatment for diseases. But even in the 1970s, almost 25% of all prescription pharmaceuticals given out in the United States were made from plants, and by 2000, that percentage had increased to 50% [2]. However, excessive use of synthetic drugs leads to a variety of serious or adverse reactions. In the United States of America, these effects are responsible for about 8% of hospital stays [3]. 

Despite the considerable advances in technology, science, and medicine during this era, we are still unable to control the exponential spread of infectious diseases and, according to the World Health Organization (WHO, Geneva, Switzerland), infections remain the second leading cause of death in the world [4]. Antibiotics, first derived from nature and then transformed into synthetic or semi-synthetic drugs, remain crucial tools in the fight against infectious diseases, but the effects of the use of broad-spectrum antibiotics on the stability of the microbiome and the resistance of pathogens are prompting studies on more selective solutions [5]. Moreover, infectious diseases are considered as major risk factors for several pathologies such as cancer, diabetes, and autoimmune and chronic inflammatory diseases; therefore, the use of antimicrobial agents reduces risk factors associated with these pathologies. 

Currently, different phytochemical substances found in medicinal plants have different physiological effects on humans and may be used to develop new drugs with various pharmacological properties [6,7]. Therefore, scientific studies on antimicrobials and anti-inflammatory drugs from plants and natural sources are of paramount importance. Indeed, drug research is oriented towards natural resources, in particular medicinal plants which have a diversified secondary metabolism with several chemical classes such as polyphenols, flavonoids, and EOs [8,9,10,11,12,13].

Recently, natural plants and their derivatives have been used to discover alternative bioactive substances that can be used as supplements or food additives; due to the presence of ethanol, common beer typically has a low concentration of bioactive molecules. Enhancing beer with various flavonoids and other dietary supplements such as taurine, resveratrol, and caffeine has increased its nutritional value [14]. The common bean (*Phaseolus vulgaris*) has been shown to have significant anti-obesity potential due to its high phenolic content and antioxidant effects, exhibiting significant effectiveness against fat formation and insulin resistance [15]. According to recent studies, many food wastes, especially the byproducts of vegetables and fruits, are an excellent source of bioactive substances that may be isolated and added to food as natural food additives or used as complex matrices to create nutraceuticals and functional meals [16].

EOs are volatile compounds synthesized and secreted by aromatic medicinal plants. They have demonstrated a range of biological and pharmacological activities such as antidiabetic, anticancer, and antiparasitic effects [17,18,19,20,21,22,23,24,25]. 

Among the medicinal plants that secrete EOs are species of the Lamiaceae family. *Origanum compactum* Benth. (*O. compactum*) is among the most widely used medicinal plants of the Lamiaceae family in traditional medicine for the treatment of several diseases, in particular microbial infections and diarrhea [26,27]. OCEO has shown tremendous biological effects, such as antimicrobial, antioxidant, antifungal, anti-inflammatory, antibacterial, antimutagenic, cytotoxic, and anticancer activities. These activities are mainly due to the major compounds of *O. compactum* such as carvacrol, thymol, and *p*-cymene [28,29]. Although, the biological activities of *O. compactum* have been evaluated by several investigations. However, these effects are variable depending on the chemical composition, which also depends on the region of collection.

As *O. compactum* is one of the most appreciated aromatic herbs, widely used in Moroccan folk medicine, this work was undertaken in order to determine the chemical composition of the EO of *O. compactum* collected from two different regions (Boulemane and Taounate), and then we assessed its biological activity, namely the antioxidant, anti-inflammatory, and antimicrobial effects.

## 2. Results and Discussion

### 2.1. Yeild and Chemical Composition of O. compactum Essential Oil

Essential oils obtained from the aerial part of O. compactum from the Boulemane and Taounate regions yielded 1.15 and 0.97% (*v*/*w*), respectively. Table 1 presents the chemical components of OCEO from the Boulemane and Taounate regions, along with characteristics of each compound’s % content, elution order, structural subclass, and retention index. As can be seen, OCEO of the Boulemane and Taounate regions revealed the presence of 11 chemical compounds, which represent 96.45% and 58.15% of the total composition of these EOs, respectively. The monoterpene constitutes the most important fraction of OCEO from Boulemane (monoterpene hydrocarbons 26.94%, oxygenated monoterpenes 66.43%). The EO from Boulemane is dominated by carvacrol (45.80%), followed by thymol (18.86%) and α-pinene (13.43%), contrary to OCEO from Taounate, which had a significant level of 3-carene (19.56%), accompanied by other constituents with variable contents, such as thymol (12.98%) and o-cymene (11.16%).

Numerous investigations examined the chemical components OCEO in various Moroccan districts, including the province of Ouezzane (northwest of Morocco) [30], which contains carvacrol, thymol, *p*-cymene, and *γ*-terpinene as major components, with 37 components represented mainly by oxygenated monoterpenes (49.4–62.975%) and hydrocarbons monoterpenes (31.815–43.632%). However, OCEO of the Rabat region is dominated by carvacrol (35.2%) and *γ*-terpinene (20.1%) as chemotypes [31]. 

In Chefchaouen (Rif region) [32], twelve compounds have been identified in OCEO wild plants. Carvacrol was the predominant compound (59.0%), followed by *p*-cymene (18.4%) and then *γ*-terpinene (8.4%). The same species from the Taounate region of Northern Morocco has demonstrated that EO has a considerable amount of certain chemical components, which should be mentioned. Twenty-six constituents were characterized with carvacrol (43.97%), *p*-cymene (17.87%), and thymol (11.56%) as the major components [33]. 

Nonetheless, our results are compatible with those of previous studies on OCEO from the Meknes region [34] (characterized by the predominance of thymol (56.41%) and (+)-3-carene (13.56%)). In addition, among the different classes of EOs are ten monoterpene hydrocarbons, a sesquiterpene hydrocarbon, four oxygenated monoterpenes, and two oxygenated sesquiterpenes representing, respectively, 27.18%, 3.67%, 57.21%, and 0.66% of the chemical composition. 

It has been noted that the chemical profiles of EOs should vary due to the direction of biosynthesis toward the preferential development of certain products as a consequence of the influence of the seasons, plant age, ecological parameters such as climatic conditions (humidity, temperature), time of collection, and geographic origin [35,36]. Effectively, a considerable impact of environmental parameters (K_2_O content, pH, and soil texture) and variability in EO productivity of the same species of *O. compactum* has been evidenced in the study conducted by Aboukhalid [37]. Furthermore, in a total of 36 plant specimens from indigenous species of *O. compactum* gathered during flowering, EO renderings have drastically differed from 0.31% to 2.44% of dry matter. However, there was no significant correlation between yield and harvest area altitude, pH of the soil, and K_2_O or P_2_O_5_ levels [38].

### 2.2. Antioxidant Activity

The methods used to determine the antioxidant activity of medicinal plants have made remarkable progress in the past few decades. However, each antioxidant activity is quantitatively classified by its mechanism of action by which the applied compounds stop chain-breaking reactions. As shown in Table 2, both EOs showed significant antioxidant activity with significant variability between the used methods: the tested essential oil 1 (EO1) from the Boulemane region exhibited a strong effect in terms of antioxidant properties with values of IC_50_ = 0.27 ± 0.01 mg/mL and IC_50_ = 0.19 ± 0.03 mg/mL attained by DPPH/FRAP assays, respectively, and an IC50 equal to 0.11 ± 0.01 mg/mL was obtained by lipid peroxidation assay. A promising DPPH radical scavenging potential effect was observed also with the tested essential oil 2 (EO2) from the Taounate region (IC_50_ = 0.37 ± 0.03 mg/mL), and an IC_50_ equal to 0.11 ± 0.01 mg/mL was obtained by FRAP assay. The EO 2 sample is also the most active in the protection from lipid peroxidation, with an IC_50_ value of 0.19 ± 0.03 mg/mL. The IC_50_ values of the tested Eos from the three antioxidant activity assays are significantly higher than that of the tested BHA standard (0.2 ± 0.01, 0.04 ± 0.05, and 0.03 ± 0.01 mg/mL, respectively). The high antioxidant activity of essential oil of *O. compactum* has already been indicated in several works including Jeldi et al. [32]. These results can probably be attributed to the phenolic compounds present in the two oils [39]. However, this effect does not reflect a single phenolic constituent. Certainly, it is linked to their richness in oxygenated monoterpenes (66.43% and 15.86%) and in monoterpene hydrocarbons (26.94% and 40.64%) [27]. As explained by Rice-Evans et al., there is a strong correlation between the antioxidant properties and the contents of the phenolic compound which allows them to act as reducing agents, hydrogen donors, singlet oxygen extinguishers, metal chelators, and facilitates lipid peroxidation. In addition, monoterpenic EOs are considered natural antioxidants [40]. This may lead to establishing a relationship between the phytoconstituents present in the EOs and the possible effect on the antioxidant capacity, essentially represented by the ability to catalyze the production of reactive oxygen species (ROS). Taking into account the fact that the major compounds of the two EOs from *O. compactum* are caryophyllene and thymol, which collectively have well-documented potent antioxidant activity [40], a study conducted by Sarikurkcu et al. [41] reported that thymol showed the strongest DPPH-scavenging activity. In addition, Foti and Inglod, [42] reported that terpenes and caryophyllene possessed a potent antioxidant capacity for preventing lipid peroxidation. In our work, caryophyllene, *α*-phellandrene, *p*-cymene, and *α*-pinene may contribute to the antioxidant properties of the OCEOs wherein the potential of synergism may occur in the EOs.

In general, therapeutic approaches using free radical scavengers (antioxidants) have shown promise in preventing, retarding, managing, or improving many complex diseases, especially neurodegeneration and cardiovascular diseases. As evidenced in different research, antioxidants may play a significant role in preventing or reducing cellular damage and further alterations occurring in cells, such as dysfunction of mitochondria, mutations in DNA, and lipid peroxidation in the cell membrane [43,44]. ROS are omnipresent signaling molecules in biological systems. In humans, a deficiency of ROS provokes excessive and persistent microbial infections, whereas an unregulated delivery of these factors causes diseases due to over-inflammation. Professional phagocytes such as eosinophils, neutrophils, macrophages, and monocytes use superoxide-generating NADPH oxidase as a component of their antimicrobial mechanism arsenal to generate elevated levels of ROS [45]. 

All the values are mean ± SD (SD: standard deviation); all results are expressed as mg/mL. DPPH: free radical-scavenging activity method; RP: reducing power activity method; LP: inhibition of lipid peroxidation activity method.

### 2.3. In Vitro Dermatoprotective and Anti-Inflammatory Effects

As skin ages, the dermis loses its tensile strength, and roughness, dryness, and anomalies including hypo- or hyper-pigmentation also emerge. Tyrosinases play a major part in melanin formation in all aspects of life. Tyrosinase inhibitors are used to prevent serious skin disorders and skin-whitening creams [46]. The first two stages of mammalian melanogenesis are catalyzed by the targeted enzyme. The in vitro inhibition was assessed as an anti-tyrosinase assay (Table 3) to assess the dermatoprotective impact of our investigated oils. Table 3 summarizes the inhibition of EO from the Boulemane and Taounate regions. To compare the obtained results, the IC_50_ values of the enzyme inhibition were calculated as summarized in Table 4; EO1 from Boulemane showed higher inhibition than EO2 from Taounate, and the IC_50_ values were 27.51 ± 0.03 μg/mL and 41.83 ± 0.01 μg/mL, respectively. Unexpectedly, the EO from Boulemane showed more effectiveness in comparison to the reference substance utilized in our test (quercetin). The IC_50_ for quercetin was 39.62 ± 0.05 μg/mL. 

To our knowledge, no study has previously shown the dermatoprotective effects of *O. compactum* oils. With an IC_50_ of 55.13 ± 1.01 μg/mL, *O. compactum* oil tyrosinase inhibition in this investigation was higher than that of a species (*Mentha viridis*) from the same family (Lamiaceae family) [20]. Our research showed that OCEO from two regions exhibits significant anti-lipoxygenase and anti-tyrosinase actions. 

Tyrosinase inhibition is an effective tool for protecting against skin damage, as evidenced in several investigations. In the study performed by Kolbe L. et al., the in vivo (clinical trials, the appearance of age spots was visibly diminished within 8 weeks) and in vitro (melanoDerm skin model culture, IC_50_ = 13.5 μmol/L) outcomes have shown that 4-n-butylresorcinol is a very efficient tyrosinase inhibitor for the topical treatment of hyperpigmentation [47]. Furthermore, Pintus et al. [48] in their recent findings demonstrated that *Euphorbia characias* possesses a very interesting anti-aging activity. A leaf ethanolic extract of this plant was found to exhibit an inhibitory activity on tyrosinase, thereby providing a photo-protective effect on the skin [48]. Moreover, conditioned media derived from *Bifidobacterium. lactis* have the potential to protect against cellular damage associated with skin aging processes due to its power to inhibit tyrosinase activity [49].

#### Lipoxygenase Inhibition Assay

LOXs represent the most vital class of oxidative enzymes containing a non-heme iron atom in their active site. They contribute to the regulation of inflammatory responses by producing pro-inflammatory (leukotrienes) or anti-inflammatory (lipoxins) mediators [50,51]. In fact, the overexpression of LOXs and their pro-inflammatory products, leukotrienes, has been in many acute and chronic inflammatory diseases in humans. In this sense, there is a wide variety of natural molecules that have been identified to regulate the activity of the LOX enzyme and eventually yield new anti-inflammatory drugs [52]

The results of the 5-LOX inhibitory activity of the tested EOs from the Boulemane region and EO from the Taounate region were obtained as previously described in Table 3 and were found to be an important activity from Boulemane with an IC_50_ equal to 0.68 ± 0.02 µg/mL and an IC_50_ equal to 1.33 ± 0.01 µg/mL from EO from Taounate. The IC_50_ value of positive control quercetin was 0.29 ± 0.03 µg/mL. In comparison with similar work, *O. compactum* from the Tetouan-Tanger region has a reduced IC_50_ equal to 123.60 ± 5.37 µg/mL [53]. The immune system is naturally stimulated during inflammation to protect the body from harmful or external stimuli such as infection and oxidative stress. However, chronic inflammation has the potential to lead to a variety of illnesses, such as autoimmune and neurological disorders, cancer, cardiovascular disease, atherosclerosis, diabetes, and obesity [54].

### 2.4. In Vivo Anti-Inflammatory Activity

The results of the effect of the OCEOs on carrageenan-induced edema are shown in Table 4.

**Figure 1:**
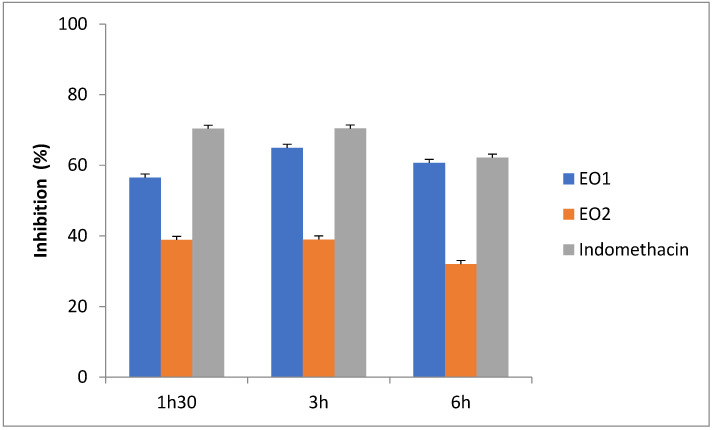
Effects of indomethacin, *O. compactum* essential oils on carrageenan induced rat paw edema.

The OCEOs from the Boulemane and EO2 Taounate regions exhibited significant (*p* < 0.05) anti-inflammatory activity as compared to the control and standard group (Table 1). At 1 h 30 min, the extract of the essential oils from Boulemane and EO from Taounate showed inhibition of edema by 56.53% and 38.86%, respectively, as compared to the standard drug indomethacin with 70.34%. However, at three hours the OCEOs from Boulemane and EO1 from Taounate showed greater inhibition with 64.95% and 39.00%, respectively, as compared to the reference drug indomethacin with 70.40% during the same time (Table 4 and Table 5; Figure 1). The most widely used approach for determining what natural medicines cause inflammation is carrageenan-induced paw edema. The biphasic event is the carrageenan-induced edema in the rat paw [55]. The initial phase, which manifests between 0 and 3 h after the injection of the phlogistic drug, has been related to the influence of mediators on vascular permeability, including histamine, serotonin, and bradykinin [55]. According to reports, the first 1.5 h following carrageenan injection are when histamine and serotonin are predominantly produced, whereas the next 2.5 h are when bradykinin is released [56]. the late phase, which may occur between three and six hours after the injection of carrageenan, is attributed to the release of prostaglandins [56].

### 2.5. Antimicrobial Activity

**Figure 2:**
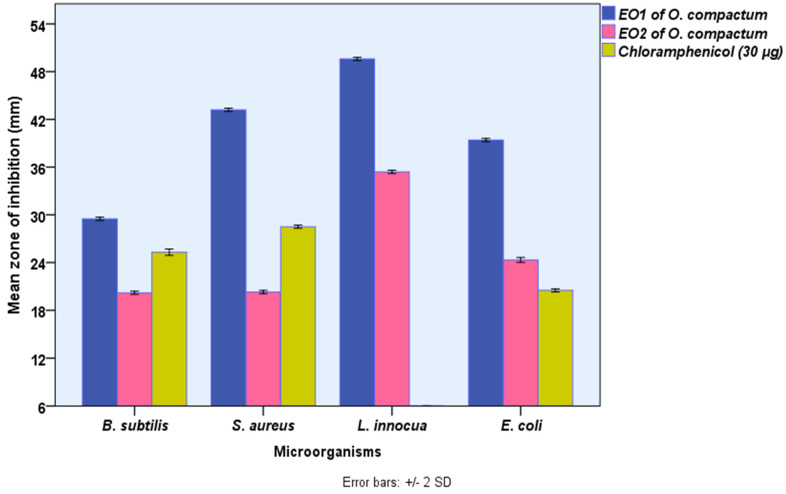
The antibacterial activities *O. compactum* EO from Boulemane region (EO1) and *O. compactum* EO from Taounat region (EO2) (paper disk zone ≤ 6.0 mm was ruled out).

The disk-diffusion assay was used to assess the potential antibacterial activity of EOs of *O. compactum* grown in the Boulemane region (EO1) and Taounate region (EO2) in vitro, and the value was computed as a mean of three replicates. As shown in Figure 2, both EOs revealed significant (*p ≤* 0.05) antibacterial activity against all tested bacteria. However, EO from Boulemane showed antibacterial activity higher than EO2 from Taounate and referenced an antibiotic using the disk-diffusion test. The measured zone of inhibition of the disk-diffusion test showed that the most susceptible bacteria against EO from Boulemane and EO2 from Taounate were *Listeria innocua* (49.6 ± 0.1, 35.4 ± 0.10 mm), followed by *Staphylococcus aureus* (43.2 ± 0.1, 20.3 ± 0.1 mm), *Escherichia coli* (39.4 ± 0.1, 24.3 ± 0.1 mm), and *Bacillus subtilis* (29.5 ± 0.1, 20.2 ± 0.1 mm), respectively. In recent decades, natural extracts have been increasingly recommended as effective substitutes for synthetic chemicals of the same strength in the food, aromatherapy, and nutraceutical sectors as well as in medicine.

The antibacterial effects of many EOs are extensively discussed in the literature [57]. Our findings are in harmony with previous studies regarding the antibacterial potential of OCEO. The latter has been reported to record remarkable antibacterial activity (in vitro) against a panel of standard reference bacteria using diffusion assays [31,39,58], and this EO was more potent as an antibacterial agent than its aqueous extract [59]. It is very interesting that a comparable investigation using the same bacterial strains from fourteen geographically distinct places across six regions in Northern Morocco was conducted several years ago and revealed some differences between the antibacterial potentials of OCEO samples [36]. Therefore, based on our current and prior investigation, we can affirm that the contents of an herb’s essential oils are influenced by environmental conditions and geographical location, and as a result, the degree of antibacterial activity of a plant is also influenced [16].

On the other side, as shown in Table 6, according to the MIC ratios, the lowest concentration of OCEO from the Boulemane area that inhibits the visible growth of bacteria varied from 0.06 to 0.25% (*v*/*v*), whereas the corresponding range for the Taounate region ranged from 0.13 to 0.21% (*v*/*v*). Additionally, the MBC ratios demonstrated that the MIC ratios for the Eos of both plants were comparable to the lowest concentration of Eos required to kill the tested bacteria in an in vitro condition. In order to understand the antibacterial mechanism of this plant’s Eos, the MBC/MIC was calculated, and an MBC/MIC ratio of 4.0 or less indicates that an antibacterial EO is bactericidal; MBC/MIC values of 4.0 or higher reveal that an antibacterial EO is bacteriostatic [60]. Accordingly, from our study, the MBC/MIC values of all tested bacteria were found to be 1.0, showing that OCEO from the two regions has noticeable bactericidal activity. Additionally, the disk-diffusion findings were corroborated to some extent by the MIC and MBC results. Our findings here are also in agreement with a previous study on the antibacterial activity of the EOs of this plant, which showed that the MIC values were typically equal to the MBC ratios [39]. The current study also revealed that *B. subtilis* and *E. coli* MIC and MBC values indicated that EO2 from Taounate was more effective than EO1 from Boulemane for these two bacteria, and this could be attributed to the phytochemical constituents of the Eos. We suggest that EOs of this plant interact synergistically with the procaryotic cell; such observation are frequently repeated in the literature [6,61,62]. Therefore, more in-depth studies on the mode of action on EOs of *O. compactum* are recommended. To understand the antibacterial mechanism of this plant, a previous study reported that the quorum-sensing phenotype of bacteria is suppressed by OCEOs, which also promote membrane permeability and disrupt cell membrane integrity [63].

### 2.6. Molecular Docking

Residues GLY116, ILE100, LEU118, ASN102, PHE88, ASN86, LEU68, TYR82, LEU53, PHE55, MET39 and ILE21 form Van der Waals interactions. There are bonds between PHE35 and the T-shaped cation pi–pi of the active site via the electron cloud flowing at the phenyl. Thus, the figure proves the high stability of the complex due mainly to the alkyl and pi-alkyl bonds formed between the VAL37 and VAL80 ligand. Therefore, it can be concluded that the obtained results illustrate the consistency with the already detailed interpretation.

## 3. Materials and Methods

### 3.1. Plant Samples and Extraction

Aerial parts of *O. compactum* (leaves, flowers, and stems) were harvested in June 2021, respectively, from Boulemane and Taounate regions (Morocco). The plants were identified according to the procedure described by González-Tejero et al. [64] and confirmed by the botanists at the Botany Department of the Scientific Institute of Rabat, University of Mohammed V Rabat, Morocco. Voucher specimens of each plant were deposited in the herbarium under the voucher specimen code RAB14812 for Boulemane and RAB13878 for the Taounate region. Then, an amount of 100 g of dried flowering tops (a mixture of leaves, flowers, and stems) of *O. compactum* was subjected to hydrodistillation for three hours using the Clevenger type device. Each extraction assay was performed with three replicates, and the recovered EO was separated from the aqueous phase using a separating funnel. The EO thus obtained was dehydrated with anhydrous sodium sulphate, weighed, and then stored at 4 °C until use in the upcoming experiments.

### 3.2. Chemical Composition Analysis of O. compactum Essential Oils

Gas chromatography coupled with mass spectra was used for chemical components analysis of OCEO by using the same conditions as described by Al-Mijalli et al. [6].

Briefly, a Hewlett-Packard (HP6890) GC instrument (Santa Clara, CA, USA) coupled with an HP5973 MS and equipped with a 5% phenylmethyl silicone HP-5MS capillary column (30 m × 0.25 mm × film thickness of 0.25 μm) was used in GC analysis. The used column temperature increased from 50 °C for 5 min to 200 °C with a 4 °C/min rate. Helium with a 1.5 mL/min flow rate and a split mode (flow: 112 mL/min; ratio: 1/74.7) was the used carrier gas. The hold time was 48 min, and the injector and detector were both 250 °C. The machine was led by a computer system of type “HP ChemStation”, managing the functioning of the machine and allowing us to follow the evolution of chromatographic analyses. Diluted samples (1/20 in methanol) of 1 μL were injected manually. In addition, 70 eV ionization voltage, 230 °C ion source temperature, and a 35–450 (*m*/*z*) scanning range were the MS operating conditions. Finally, the identification of different compounds was carried out by the comparison of MS spectra with the library and matching the Kovats index (Library of NIST/EPA/NIH MASS SPECTRAL LIBRARY Version 2.0, 1 July 2002). The quantification of the different compounds was obtained by internal normalization of the total area of peaks detected in each chromatogram

### 3.3. Antioxidant Activity Assays

#### 3.3.1. Free Radical Scavenging Ability (DPPH Method)

The ability of EO to scavenge the DPPH radical was estimated by measuring the IC_50_ value of the samples [65]. One milliliter of EO was added to 0.25 mL of DPPH solution (0.2 mmol/L (*v*/*v*)). The absorbance was determined at 517 nm after 30 min. Butylated hydroxyl anisole (BHA) was used as a positive control. All experiments were carried out in triplicate. The DPPH radical scavenging activity was calculated according to the following formula:

(1) $$\mathrm{DPPH}\;\left(\%\right)=\frac{\left(\mathrm{Abs}\;\mathrm{DPPH}-\mathrm{Abs}\;\mathrm{Sample}\right)}{\mathrm{Abs}\;\mathrm{DPPH}}\times 100\;$$

#### 3.3.2. Ferric-Reducing Antioxidant Power (FRAP) Assay

The reducing power of *O. compactum* EOs was determined according to the protocol of Singh et al. [66]. In test tubes, 1 mL of EOs was diluted in methanol at different concentrations (0.1, 0.2, 0.3, 0.4, and 0.5 mg/mL). Then, each tube was mixed with 1 mL of phosphate buffer and 1 mL of 1% of potassium ferrocyanide K3Fe(CN)_6_. After the incubation at 50 °C for 20 min, 1 mL of 10% trichloroacetic acid (TCA) was added to the mixture, followed by centrifugation at 3000 rpm for 10 min. Afterwards, the supernatant was mixed with 1.5 mL of distilled water and 150 µL of 0.1% FeCl_3_. The absorbance was measured at 700 nm and compared against BHA, which was used as the reference. The antioxidant power was expressed as an IC_50_ value (mg/mL). All samples were performed in triplicate.

#### 3.3.3. Inhibition of Lipid Peroxidation

The anti-lipid peroxidation capacity was determined by the linoleic acid/β-carotene assay, as described by Tepe et al., with slight modifications [67]. A solution of β-carotene and linoleic acid was prepared as follows: 0.5 mg of *β*-carotene was dissolved in 1 mL of chloroform and 25 μL of linoleic acid, and 200 mg of Tween 40 was added. Chloroform was evaporated. Then, 100 mL of distilled water was added. Afterwards, 2.5 mL of aliquot of this reaction mixture was dispensed into test tubes, and 350 μL of the prepared samples at 2 mg/mL was added. The emulsion was incubated for 48 h at room temperature. The same procedure was repeated with BHA, as a standard, and a blank (without BHA). After 48 h, the absorbance was measured at 490 nm. The antioxidant activity was expressed as an IC_50_ value (mg/mL). All samples were carried out in triplicate.

Relative antioxidant activity was calculated in the following way:

(2) $$\mathrm{Antioxidant}\;\mathrm{activity}\;\left(\%\right)=\frac{\mathrm{At}}{A0}\times 100$$

where A0 is the absorbance of the tested EOs at the beginning of incubation, and At is the absorbance of the tested EOs at the end of incubation.

### 3.4. In Vitro Anti-Inflammatory and Dermatoprotective Assays

The determination of dermatoprotective effect of OCEO was carried out in vitro using tyrosinase inhibitory activity assay as described by Bouyahya et al. [27] Moreover, 5-LOX inhibitory activity of OCEO was used to evaluate the in vitro anti-inflammatory effect according to the previous published method [27,40]. Briefly, 20 µL of OCEO and 20 µL of 5-LOX from Glycine max (100 U/mL) were pre-incubated with 200 µL of phosphate buffer (0.1 M, pH 9) at room temperature for 5 min. After that, 20 µL of linolenic acid (4.18 mM in ethanol) was added to the mixture (followed for 3 min at 234 nm). Each quercetin served as positive control, and the test assay was performed in triplicate.

### 3.5. In Vivo Anti-Inflammatory Assay

The in vivo anti-inflammatory effect was carried out using a rat model of carrageenan-induced paw edema [68]. Briefly, Wistar rats (160 to 190 g) were fasted for 18 h and then randomly divided into four groups containing six animals. The first two groups received orally a dose of 100 mg/kg of EO of *O. compactum*, respectively. The 3rd group was a negative control who received distilled water, while the last group was considered a positive control and received indomethacin (10 mg/kg) as the reference anti-inflammatory drug. After 60 min, all rats were injected subcutaneously with carrageenan solution (0.05 mL of 1% carrageenan suspended in 0.9% NaCl) into the sub plantar region of the left hind paw. The volume changes of both legs for each rat are measured using a plethysmometer (LE 7500 Digital) at 30 min after the initiation of inflammation and then at 1 h 30, 3 h, 4 h, 5 h, and 6 h 00 after edema induction [69]. Anti-inflammatory activity is assessed by calculating the percentage of edema inhibition (% INH) in paw volume, as follows:

%INH = (mean [VL-VR] _Control_ − mean [VL-VR] _Treated_)/[VL-VR] _Control_ × 100 (3)

where VL is volume of the left paw, and VR is volume of the right paw.

### 3.6. Antibacterial Activity

#### 3.6.1. Bacterial Strains

The antibacterial activity of OCEO was investigated against four microorganisms: *Escherichia coli* ATCC 25922, representing Gram-negative bacteria, and *Bacillus subtilis* ATCC 6633, *Staphylococcus aureus* ATCC 29213, and *Listeria innocua* ATCC 33090, representing Gram-positive bacteria.

#### 3.6.2. Growth Conditions

A loopful of the frozen stock (−20 °C) was used to inoculate Mueller-Hinton Agar (Biokar, Beauvais, France), and bacteria were revived by incubating them there for 24 h at 37 °C. After that, an inoculum from a bacterial colony was taken and adjusted to 0.5 McFarland in sterile saline water (0.9 % NaCl), and it was then transferred into a sterile tube where the heavy particles were allowed to settle for 5 min. The top homogenous suspensions were transferred to a fresh, sterile tube and microscopically adjusted to a concentration of 10^4^ CFU/mL. The antibacterial testing directly used the adjusted bacterial inoculum.

#### 3.6.3. Disk-Diffusion Assay

The disk-diffusion technique was used to determine the primary screening of the examined EO antibacterial activity in accordance with the previously published procedures [70,71]. In brief, the culture suspension was inoculated by swabbing on Mueller-Hinton Agar medium (Biokar, Beauvais, France). Then, sterile paper disks measuring 6 mm in diameter were placed on each plate and soaked with 10 µL of each EO (combined with 5% of DMSO). The positive control for bacteria was chloramphenicol (30 µg), while the negative control was DMSO (10 µL; 5%). The bacterial plates underwent a 24 h incubation period at 37 °C. The findings were presented as the mean ± standard deviation of three repetitions, and the inhibitory diameters were measured in millimeters after incubation.

#### 3.6.4. Determination of MIC

The MIC corresponds to the minimum concentration of EO that can inhibit the growth of microorganisms. In fact, the determination of MIC values against bacteria was performed according to the protocol described previously, with some modifications [72], in which Mueller-Hinton broth (Biokar, Beauvais, France) was used with bacteria. The incubation was conducted at 37 °C for 24 h. Chloramphenicol was used as a positive control. Twofold serial dilutions of EO concentrations ranging from 4% to 0.0625 percent (*v*/*v*) were prepared in sterile microtubes. Then, 4 µL of the bacterial suspensions that had already been prepared were added to each tube. All suspensions were homogenized and incubated at 37 °C for 24 h. The liquid medium without EOs that had been inoculated with bacterial suspensions served as the positive control. As a negative control, liquid medium and EO-filled microtubes were uninoculated. After being incubated, the MIC value was observed in microtubes with low concentrations of bacteria and no visible growth of bacteria [60].

#### 3.6.5. Determination of MBC

The MBC test was carried out by subculturing 10 µL from a microtube on the growth medium that did not exhibit bacterial growth and then incubating the plates overnight at 35–37 °C. It was established that the MBC was the lowest concentration, at which there was no growth in the medium. Chloramphenicol was used as the reference test [7]. Moreover, MBC/MIC was calculated to understand the possible mechanism of the tested compound [73].

### 3.7. Molecular Docking Studies

Molecular docking was performed to predict the interaction of carvacrol (main bioactive compound of OCEO) with the active site of DNA-gyrase [1,74]. The crystal structure of the enzyme (PDB code: 1KZN, 2.3 Å) was chosen as the protein target for this study. The structure of the methionine (ligand) was optimized using HyperChem 8.0.10 software to determine the amino acids responsible for the active sites [2,75]. Afterwards, we performed docking for the most predominant compound, such as carvacrol.

Auto Dock tools were used to prepare the molecules and parameters before submitting them to docking analysis with Auto Dock [2,75,76]. Polar hydrogen atoms were added while fusing non-polar hydrogen atoms, and then Gasteiger partial atomic charges were assigned to the ligands. The prepared protein and ligand structures were saved in PDBQT format intended for the calculation of energy grid maps. A grid box size of 50 × 50 × 50 Å points with a grid spacing of 0.375 Å was considered.

**Figure 3:**
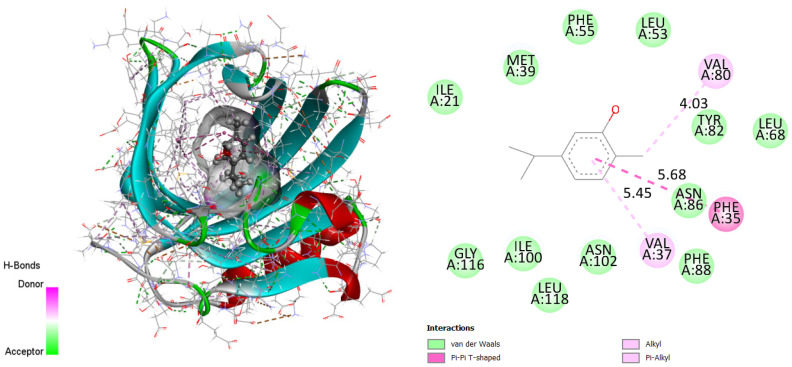
Two-dimensional and three-dimensional docking poses proving carvacrol interactions in the binding site (binding energy 7.04 kcal/mol). This figure was made with Discovery Studio 3.5.

The Lamarckian Genetic Algorithm (LGA) program with a full adaptive search method in Auto Dock was chosen to calculate the different ligand conformers. After 200 independent docking trials for the ligand, based on the root mean square deviation (RMSD) tolerance of 2.0 Å, the conformation chosen was the one with the minimum energy [4,77] (Figure 3).

### 3.8. Statistical Analysis

One-way ANOVA was used to verify the statistical significance of the data. At a significance level of *p* ≤ 0.05, differences were deemed statistically significant. SPSS software package (IBM SPSS statistics, v.23) was used in the statistical analysis.

## 4. Conclusions and Perspectives

*Origanum compactum* is a traditional herbal medicine used by Moroccan populations to treat several diseases. Chemical investigations have proven that this plant species contains many bioactive components, especially volatile molecules. There is still a need for further research into the discovery of additional bioactive ingredients such as aromatic flavonoids, alkaloids, and phenolics but also concerning their toxicity, in order to monitor their safety. In addition, through in vitro data, *O. compactum* has been found to display numerous pharmacological properties such as anti-inflammatory, antimicrobial, dermatoprotective, antioxidant, anticancer, and antifungal effects, which explains its effective application in the tradition. In this work, we have proven for the first time in Morocco that this plant originating from the studied regions (Boulemane and Taounate) exhibits an excellent dermatoprotective effect. Both EOs showed remarkable antibacterial, anti-fungal, antioxidant, and anti-inflammatory activities. However, further investigations should be conducted concerning pharmacodynamics as well as pharmacokinetics pathways in order to explore single molecules or mixtures for stimulating activities that can be directed in the health, pharmaceutical, or food industry and agriculture.

## Figures and Tables

**Table 1:** Chemical composition of *O. compactum* essential oils.

	EO1 Boulemane			EO2 Taounate		
Number	RT	Compounds	%	RT	Compounds	%
**1**	2.014	*α*-Thujene	0.30	4.218	*α*-Thujene	1.43
**2**	2.081	*α*-Pinene	0.79	4.353	*α*-Pinene	0.93
**3**	3.219	*ß*-Myrcene	1.85	4.624	Camphene	0.14
**4**	3.298	*α*–Phellandrene	0.30	5.187	*β*-Pinene	0.39
**5**	3.591	(+)-4-Carene	2.28	5.503	*β*-Myrcene	0.39
**6**	3.873	*p*-Cymene	**7.99**	5.773	*α*-Phellandrene	0.41
**7**	4.729	*β*-Pinene	**13.43**	6.089	(+)-4-Carene	3.27
**8**	5.710	Linalool	1.77	6.359	o-Cymene	**11.16**
**9**	11.896	Carvacrol	**45.80**	6.416	*β*-Phellandrene	0.6
**10**	12.155	Thymol	**18.86**	7.261	3-Carene	**19.56**
**11**	13.834	Caryophyllene	3.08	7.824	*ρ*-Cymene	0.33
**12**				8.151	Linalool	1.79
**13**				13.335	Thymol	**12.98**
**14**				16.445	Caryophyllene	1.8
**Total identified** **compounds %**			**96.45**	**Total identified** **compounds %**		**58.15**
**Monoterpene hydrocarbons %**			**26.94**	**Monoterpene hydrocarbons %**		**40.49**
**Oxygenated monoterpenes %**			**66.43**	**Oxygenated monoterpenes %**		**15.86**
**Sesquiterpene hydrocarbons %**			**3.08**	**Sesquiterpene hydrocarbons %**		**1.8**
**Oxygenated sesquiterpenes %**			**---**	**Oxygenated sesquiterpenes %**		**---**

**Table 2:** Antioxidant activity of *O. compactum* essential oils.

	**IC50** **(mg/mL)**		
	EO1 Boulemane	EO2 Taounate	**BHA**
DPPH	0.27 ± 0.01	0.37 ± 0.03	0.2 ± 0.01
Ferric-Reducing (RP)	0.19 ± 0.03	0.25 ± 0.04	0.04 ± 0.05
Lipid peroxidation (LP)	0.11 ± 0.01	0.19 ± 0.03	0.03 ± 0.01

**Table 3:** In vitro anti-inflammatory and dermatoprotective activity.

Assay (IC_50_ μg/mL)	EO1 Boulemane	EO2 Taounate	Quercetin
**5-Lipoxygenase**	0.68 ± 0.02	1.33 ± 0.01	0.29 ± 0.03
**Tyrosinase**	27.51 ± 0.03	41.83 ± 0.01	39.62 ± 0.05

**Table 4:** Effect of *O. compactum* essential oils on carrageenan-induced rat paw.

Treatment Group	Mean Edema Volume (Left-Right Paw) mL		
	1 h 30 min	3 h	6 h
**Control**	0.543 ± 0.01	0.659 ± 0.04	0.534 ± 0.02
**Indomethacin**	0.161 ± 0.04 *	0.195 ± 0.06 *	0.202 ± 0.03 *
**EO1 Boulmane**	0.236 ± 0.03 *	0.231 ± 0.01 *	0.210 ± 0.01 *
**EO2 Taounate**	0.332 ± 0.02 *	0.402 ± 0.01 *	0.363 ± 0.03 *

Values are expressed as mean ± SD; SD, standard deviation; (*n* = 6 of each group). * *p* < 0.05 statically significant compared to the control. The reference drug is indomethacin.

**Table 5:** Percentage of inflammation inhibition by *O. compactum* on carrageenan-induced rat paw edema.

Treatment Group	Percentage Inhibition of Edema (%)		
	1 h 30 min	3 h	6 h
Indomethacin	70.34	70.40	62.17
EO1 Boulemane	56.53 *	64.95 *	60.67 *
EO2 Taounate	38.86 *	39.00 *	32.02 *

Values are expressed as mean ± SD; SD, standard deviation; (*n* = 6 of each group). * *p* < 0.05 statically significant compared to the control. The reference drug is indomethacin.

**Table 6:** MIC, MBC, and MBC/MIC values of *O. compactum* from Boulemane region (EO1 Boulemane) and *O. compactum* from Taounate region (EO2 Taounate).

	Gram-Positive Bacteria									Gram-Negative Bacteria		
	*B. subtilis*			*S. aureus*			*L. innocua*			*E. coli*		
	MIC	MBC	MBC/ MIC	MIC	MBC	MBC/ MIC	MIC	MBC	MBC/MIC	MIC	MBC	MBC/MIC
EO1 from Boulemane (%) *v*/*v*	0.21	0.21	1	0.12	0.12	1	0.06	0.06	1	0.25	0.25	1
EO2 from Taounate (%) *v*/*v*	0.15	0.15	1	0.15	0.15	1	0.13	0.13	1	0.21	0.21	1
Chloramphenicol (30 μg)	0.25	0.5	2	4.0	8.0	2	ND	ND	ND	8.0	8.0	1

ND: not detected.

## Data Availability

Not applicable.

## Sample Availability

Samples of the compounds are available from the authors.
